# Ethane-1,2-diyl bis­(benzene­dithio­ate)

**DOI:** 10.1107/S1600536811010245

**Published:** 2011-03-26

**Authors:** Daisuke Abe, Yuji Sasanuma, Hiroyasu Sato

**Affiliations:** a Department of Applied Chemistry and Biotechnology, Chiba University, 1-33 Yayoi-cho, Inage-ku, Chiba 263-8522, Japan; b Application Laboratory, Rigaku Corporation, 3-9-12 Matsubara-cho, Akishima-shi, Tokyo 196-8666, Japan

## Abstract

In the crystal structure, the title compound, C_16_H_14_S_4_, is located on an inversion center and exhibits a *gauche^+^*–*trans*–*gauche^−^* conformation in the S—CH_2_—CH_2_—S bond sequence. The S—C=S plane makes a dihedral angle of 30.63 (17)° with the phenyl ring. An inter­molecular C—H⋯π inter­action is observed.

## Related literature

For crystal structures and conformations of related compounds with S—CH_2_—CH_2_—S bond sequences, see: for example, Takahashi *et al.* (1968 ▶); Deguire & Brisse (1988 ▶); Sasanuma & Watanabe (2006 ▶).
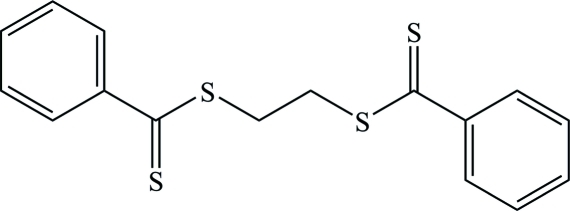


## Experimental

### 

#### Crystal data

C_16_H_14_S_4_
*M* *_r_* = 334.53
Monoclinic, 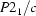

*a* = 11.5431 (7) Å
*b* = 8.74071 (16) Å
*c* = 8.93720 (16) Å
β = 122.3772 (7)°
*V* = 761.54 (5) Å^3^
*Z* = 2
Cu *K*α radiation
μ = 5.60 mm^−1^
*T* = 93 K
0.32 × 0.27 × 0.08 mm

#### Data collection

Rigaku R-AXIS RAPID diffractometer
Absorption correction: multi-scan (*ABSCOR*; Higashi, 1995 ▶) *T*_min_ = 0.195, *T*_max_ = 0.639
8415 measured reflections
1389 independent reflections
1292 reflections with *F* ^2^ > 2σ(*F* ^2^)
*R*_int_ = 0.054

#### Refinement

*R*[*F* ^2^ > 2σ(*F* ^2^)] = 0.030
*wR*(*F* ^2^) = 0.079
*S* = 1.14
1389 reflections
91 parameters
H-atom parameters constrained
Δρ_max_ = 0.31 e Å^−3^
Δρ_min_ = −0.39 e Å^−3^

### 

Data collection: *PROCESS-AUTO* (Rigaku, 1998 ▶); cell refinement: *PROCESS-AUTO*; data reduction: *CrystalStructure* (Rigaku Americas & Rigaku, 2007 ▶); program(s) used to solve structure: *SIR2004* (Burla *et al.*, 2005 ▶); program(s) used to refine structure: *SHELXL97* (Sheldrick, 2008 ▶); molecular graphics: *ORTEPII* (Johnson, 1976 ▶); software used to prepare material for publication: *CrystalStructure*.

## Supplementary Material

Crystal structure: contains datablocks global, I. DOI: 10.1107/S1600536811010245/is2686sup1.cif

Structure factors: contains datablocks I. DOI: 10.1107/S1600536811010245/is2686Isup2.hkl

Additional supplementary materials:  crystallographic information; 3D view; checkCIF report

## Figures and Tables

**Table 1:** Hydrogen-bond geometry (Å, °) *Cg*1 is the centroid of the C1–C6 phenyl ring.

*D*—H⋯*A*	*D*—H	H⋯*A*	*D*⋯*A*	*D*—H⋯*A*
C8—H8*A*⋯*Cg*1^i^	0.99	2.65	3.451 (1)	138

Symmetry code: (i) 
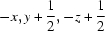
.

**Fig. 1.**
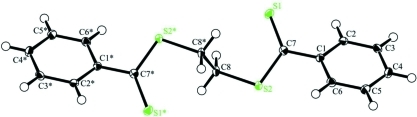
Molecular structure of 1,2-bis(dithiobenzoyl)ethane (BDTBE). Displacement ellipsoids are drawn at the 50% probability level. The asterisk corresponds to symmetry code -x, -y + 1, -z.

**Fig. 2.**
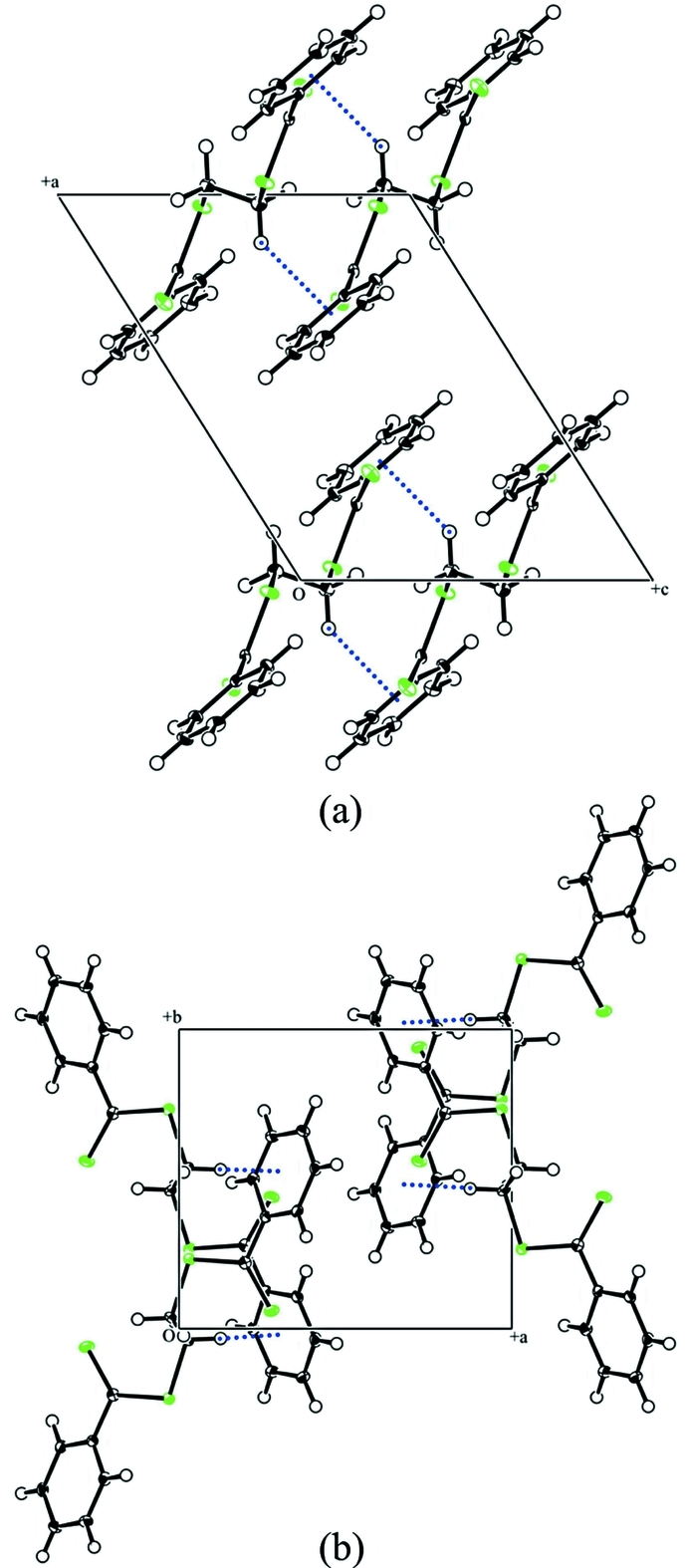
Packing diagram of BDTBE, viewed down (a) the b axis and (b) the c axis. Displacement ellipsoids are drawn at the 50% probability level. The dotted lines represent C–H···π interactions.

## supplementary crystallographic information

### Comment

The S—CH_2_—CH_2_—S part of crystallized poly(ethylene sulfide) (PES, [–CH_2_CH_2_S–]*_x_*) lies in the *gauche^+^* – *trans* – *gauche^-^* (*g*^+^*tg*^-^) conformation (Takahashi *et al.*, 1968); the two S—C bonds are in opposite *gauche* states, and dipole moments are formed along bisectors of the C—S—C angles. The dipole–dipole interaction was suggested to be the source of its high melting point (215–220 °C) in comparison with that (66–69 °C) of poly(ethylene oxide), [–CH_2_CH_2_O–]*_x_* (Sasanuma & Watanabe, 2006). Therefore, poly(thioethylenethioterephthaloyl) ([–SCH_2_CH_2_SCOC_6_H_4_CO–]*_x_*) and poly(thioethylenethiodithioterephthaloyl) ([–SCH_2_CH_2_SCSC_6_H_4_CS–]*_x_*), having the same S—CH_2_—CH_2_—S bond sequence as PES, are expected to be superior in some physical properties to their homologous polyester, poly(ethylene terephthalate) ([–OCH_2_CH_2_OCOC_6_H_4_CO–]*_x_*). Crystal conformations of polymers are requisite to derive their configurational properties and thermodynamic quantities. Because a polymer tends to have a crystal conformation similar to that of its small model compounds, the models provide the physicochemical information on the polymer. The crystal structure of 1,2-bis(benzoylthio)ethane (BBTE, C_6_H_5_C(=O)SCH_2_CH_2_SC(=O)C_6_H_5_), a model compound of poly(thioethylenethioterephthaloyl), was determined already (Deguire & Brisse, 1988); its S—CH_2_—CH_2_—S part also lies in the *g*^+^*tg^-^* state. We have investigated structure-property relationships of the above-mentioned polyester, polythioester, and polydithioester. As part of the work, this study has dealt with 1,2-bis(dithiobenzoyl)ethane (BDTBE, C_6_H_5_C(=S)SCH_2_CH_2_SC(=S)C_6_H_5_), a model compounds of poly(thioethylenethiodithioterephthaloyl); the crystal structure has been determined and compared with those of BBTE and PES.

Figure 1 shows the molecular structure of BDTBE. Its S—CH_2_—CH_2_—S bond sequence adopts the *g^+^tg^-^* conformation, as found for PES and BBTE. The *g^+^tg^-^* conformation renders the two phenyl rings parallel to each other; however, this is partly because the BDTBE molecule is located on the center of symmetry. The C_6_H_5_—C(=O)—S part of BBTE is essentially coplanar, whereas the C=S bond of BDTBE is out of the phenyl plane; the S–C═S plane makes a dihedral angle of 30.63 (17)° with the phenyl ring. This is probably due to the van der Waals radius (1.80 Å) of sulfur larger than that (1.52 Å) of oxygen.

The BBTE crystal seems to include intermolecular π–π interactions of a near vertical type (Deguire & Brisse, 1988). In addition, dipole moments, formed close to the O=C bonds, are either parallel or antiparallel to one another. The dipole-dipole interactions are known to stabilize the crystal structure (Sasanuma & Watanabe, 2006). On the other hand, Figure 2 shows that the C=S bonds of BDTBE do not have such clear orientations, because the small difference in electronegativity between C and S little polarizes the C=S bond. In the BDTBE crystal, instead, C—H···π interactions appear to exist between C8—H8A bond and its neighboring phenyl (Ph) ring, and the H···Ph spacing can be estimated to be 2.65 Å.

### Experimental

Benzoyl chloride (19.5 g) was added dropwise into 1,2-ethanedithiol (6.2 g) and pyridine (11 ml) in a four-neck flask equipped with a mechanical stirrer and a reflux condenser, and the mixture was stirred at 0 °C for 30 m and, furthermore, at room temperature overnight. The reaction mixture was subjected to extraction with water and ether. The organic layer was washed three times with 8% sodium hydrogen carbonate solution, dried overnight over anhydrous magnesium sulfate, filtrated, and condensed on a rotary evaporator. The residue was recrystallized twice from ethanol and dried under reduced pressure. 1,2-Bis(benzoylthio)ethane (1.5 g) thus prepared, Lawesson's reagent (2.5 g), and toluene (10 ml) were mixed and refluxed for 5 h. The reaction mixture was condensed, dissolved in a mixed solvent (15 ml) of toluene and *n*-hexane (volume ratio 1:3), and fractionated by a silica-gel column chromatograph. The reddish fraction was collected, condensed, recrystallized twice from ethanol, and dried *in vacuo*.

Crystals for X-ray diffraction were prepared by slow evaporation of a dimethyl sulfoxide solution. Then, the solution was kept in an open vessel so that water vapor, a poor solvent, would be immixed and hasten the crystallization.

### Refinement

All C—H hydrogen atoms were geometrically positioned with C—H = 0.95 and 0.99 Å for the aromatic and methylene groups respectively, and refined as riding by *U*_iso_(H) = 1.2*U*_eq_(C).

### Crystal data

**Table:**

C_16_H_14_S_4_	*F*(000) = 348.00
*M**_r_* = 334.53	*D*_x_ = 1.459 Mg m^−^^3^
Monoclinic, *P*2_1_/*c*	Cu *K*α radiation, λ = 1.54187 Å
Hall symbol: -P 2ybc	Cell parameters from 7723 reflections
*a* = 11.5431 (7) Å	θ = 4.5–68.2°
*b* = 8.74071 (16) Å	µ = 5.60 mm^−^^1^
*c* = 8.93720 (16) Å	*T* = 93 K
β = 122.3772 (7)°	Prism, orange
*V* = 761.54 (5) Å^3^	0.32 × 0.27 × 0.08 mm
*Z* = 2	

### Data collection

**Table:**

Rigaku R-AXIS RAPID diffractometer	1292 reflections with *F*^2^ > 2σ(*F*^2^)
Detector resolution: 10.00 pixels mm^-1^	*R*_int_ = 0.054
ω scans	θ_max_ = 68.2°
Absorption correction: multi-scan (*ABSCOR*; Higashi, 1995)	*h* = −13→13
*T*_min_ = 0.195, *T*_max_ = 0.639	*k* = −10→10
8415 measured reflections	*l* = −10→10
1389 independent reflections	

### Refinement

**Table:**

Refinement on *F*^2^	Secondary atom site location: difference Fourier map
*R*[*F*^2^ > 2σ(*F*^2^)] = 0.030	Hydrogen site location: inferred from neighbouring sites
*wR*(*F*^2^) = 0.079	H-atom parameters constrained
*S* = 1.14	*w* = 1/[σ^2^(*F*_o_^2^) + (0.0329*P*)^2^ + 0.4332*P*] where *P* = (*F*_o_^2^ + 2*F*_c_^2^)/3
1389 reflections	(Δ/σ)_max_ < 0.001
91 parameters	Δρ_max_ = 0.31 e Å^−^^3^
0 restraints	Δρ_min_ = −0.39 e Å^−^^3^
Primary atom site location: structure-invariant direct methods	

### Special details

**Table:**

Geometry. All e.s.d.'s (except that e.s.d. in the dihedral angle between two l.s. planes) are estimated using the full covariance matrix. The cell e.s.d.'s are taken into account individually in the estimation of e.s.d.'s in distances, angles, and torsion angles; correlations between e.s.d.'s in cell parameters are only used when they are defined by crystal symmetry. An approximate (isotropic) treatment of cell e.s.d.'s is used for estimating e.s.d.'s involving l.s. planes.
Refinement. Refinement was performed with all reflections. The weighted *R*-factor (*wR*) and goodness of fit (*S*) are based on *F*^2^, while the *R*-factor on *F*. The threshold expression of *F*^2^ > 2.0 σ(*F*^2^) was used only for calculating *R*-factor.

### Fractional atomic coordinates and isotropic or equivalent isotropic displacement parameters (Å^2^)

**Table:**

	*x*	*y*	*z*	*U*_iso_*/*U*_eq_	
S1	0.27738 (4)	0.43962 (5)	0.38957 (6)	0.01677 (15)	
S2	0.03137 (4)	0.26716 (5)	0.11103 (6)	0.01529 (15)	
C1	0.26047 (17)	0.1259 (2)	0.3631 (2)	0.0107 (3)	
C2	0.35523 (17)	0.1069 (2)	0.5448 (2)	0.0132 (4)	
C3	0.41019 (18)	−0.0366 (2)	0.6127 (2)	0.0154 (4)	
C4	0.37290 (18)	−0.1615 (2)	0.4996 (2)	0.0174 (4)	
C5	0.28006 (18)	−0.1431 (2)	0.3188 (2)	0.0157 (4)	
C6	0.22359 (18)	−0.0005 (2)	0.2505 (2)	0.0134 (3)	
C7	0.19836 (18)	0.2793 (2)	0.2954 (2)	0.0116 (3)	
C8	−0.02347 (18)	0.4635 (2)	0.0565 (2)	0.0143 (4)	
H2	0.3821	0.1924	0.6221	0.016*	
H3	0.4733	−0.0493	0.7363	0.019*	
H4	0.4109	−0.2594	0.5460	0.021*	
H5	0.2552	−0.2285	0.2417	0.019*	
H6	0.1596	0.0112	0.1269	0.016*	
H8A	−0.1248	0.4677	−0.0080	0.017*	
H8B	0.0137	0.5229	0.1674	0.017*	

### Atomic displacement parameters (Å^2^)

**Table:**

	*U*^11^	*U*^22^	*U*^33^	*U*^12^	*U*^13^	*U*^23^
S1	0.0199 (2)	0.0102 (2)	0.0133 (2)	−0.00211 (16)	0.0043 (2)	−0.00110 (17)
S2	0.0121 (2)	0.0111 (2)	0.0152 (3)	0.00043 (15)	0.0024 (2)	0.00257 (17)
C1	0.0106 (7)	0.0115 (8)	0.0113 (9)	0.0003 (6)	0.0068 (7)	0.0013 (7)
C2	0.0112 (7)	0.0137 (9)	0.0129 (10)	−0.0019 (6)	0.0053 (7)	0.0001 (7)
C3	0.0113 (8)	0.0176 (9)	0.0123 (10)	−0.0007 (6)	0.0029 (7)	0.0027 (7)
C4	0.0150 (8)	0.0120 (9)	0.0236 (11)	0.0024 (6)	0.0092 (8)	0.0045 (8)
C5	0.0162 (8)	0.0118 (8)	0.0168 (10)	−0.0003 (6)	0.0072 (8)	−0.0021 (7)
C6	0.0127 (8)	0.0156 (9)	0.0098 (9)	−0.0005 (6)	0.0045 (7)	0.0005 (7)
C7	0.0134 (8)	0.0137 (9)	0.0089 (9)	0.0001 (6)	0.0067 (7)	0.0002 (7)
C8	0.0143 (8)	0.0119 (8)	0.0153 (10)	0.0050 (6)	0.0070 (8)	0.0039 (7)

### Geometric parameters (Å, °)

**Table:**

S1—C7	1.6376 (17)	C5—C6	1.387 (2)
S2—C7	1.7436 (15)	C8—C8^i^	1.519 (3)
S2—C8	1.8033 (17)	C2—H2	0.950
C1—C2	1.400 (2)	C3—H3	0.950
C1—C6	1.399 (2)	C4—H4	0.950
C1—C7	1.488 (2)	C5—H5	0.950
C2—C3	1.390 (2)	C6—H6	0.950
C3—C4	1.389 (2)	C8—H8A	0.990
C4—C5	1.390 (2)	C8—H8B	0.990
			
S1···H4^ii^	2.990	H4···S1^vi^	2.990
C1···H8A^iii^	2.866	H4···H2^v^	2.664
C2···H8A^iii^	2.779	H5···H8A^vii^	2.763
C3···H8A^iii^	2.903	H8A···C1^viii^	2.866
H2···H3^iv^	2.687	H8A···C2^viii^	2.779
H2···H4^iv^	2.664	H8A···C3^viii^	2.903
H3···H2^v^	2.687	H8A···H5^vii^	2.763
			
C7—S2—C8	104.35 (7)	C3—C2—H2	119.9
C2—C1—C6	119.24 (15)	C2—C3—H3	120.0
C2—C1—C7	119.06 (16)	C4—C3—H3	120.0
C6—C1—C7	121.67 (14)	C3—C4—H4	120.0
C1—C2—C3	120.25 (17)	C5—C4—H4	120.0
C2—C3—C4	120.03 (16)	C4—C5—H5	119.9
C3—C4—C5	120.03 (16)	C6—C5—H5	119.9
C4—C5—C6	120.22 (17)	C1—C6—H6	119.9
C1—C6—C5	120.22 (16)	C5—C6—H6	119.9
S1—C7—S2	124.57 (10)	S2—C8—H8A	109.1
S1—C7—C1	123.21 (11)	S2—C8—H8B	109.1
S2—C7—C1	112.18 (11)	C8^i^—C8—H8A	109.1
S2—C8—C8^i^	112.39 (16)	C8^i^—C8—H8B	109.1
C1—C2—H2	119.9	H8A—C8—H8B	107.9
			
C7—S2—C8—C8^i^	83.65 (14)	C6—C1—C7—S1	−152.1 (2)
C8—S2—C7—S1	0.8 (2)	C6—C1—C7—S2	30.0 (3)
C8—S2—C7—C1	178.65 (18)	C7—C1—C6—C5	−177.9 (2)
C2—C1—C6—C5	0.3 (3)	C1—C2—C3—C4	1.1 (3)
C6—C1—C2—C3	−1.0 (3)	C2—C3—C4—C5	−0.3 (3)
C2—C1—C7—S1	29.7 (3)	C3—C4—C5—C6	−0.4 (3)
C2—C1—C7—S2	−148.13 (18)	C4—C5—C6—C1	0.5 (3)
C7—C1—C2—C3	177.1 (2)		

Symmetry codes: (i) −*x*, −*y*+1, −*z*; (ii) *x*, *y*+1, *z*; (iii) −*x*, *y*−1/2, −*z*+1/2; (iv) −*x*+1, *y*+1/2, −*z*+3/2; (v) −*x*+1, *y*−1/2, −*z*+3/2; (vi) *x*, *y*−1, *z*; (vii) −*x*, −*y*, −*z*; (viii) −*x*, *y*+1/2, −*z*+1/2.

### Hydrogen-bond geometry (Å, °)

**Table:**

*Cg*1 is the centroid of the C1–C6 phenyl ring.

**Table:**

*D*—H···*A*	*D*—H	H···*A*	*D*···*A*	*D*—H···*A*
C8—H8A···Cg1^viii^	0.99	2.65	3.451 (1)	138

Symmetry codes: (viii) −*x*, *y*+1/2, −*z*+1/2.
